# Europium Lithosilicates Li_2_EuSi_2_N_4_ and Li_2_EuSiO_4_—Crystal Structures and Luminescence

**DOI:** 10.1021/acs.chemmater.4c02070

**Published:** 2024-09-12

**Authors:** Kilian M. Rießbeck, Markus Seibald, Christiane Stoll, Hubert Huppertz

**Affiliations:** † Department of General, Inorganic and Theoretical Chemistry, University of Innsbruck Innrain 80-82, A-6020 Innsbruck, Austria; ‡ ams-OSRAM International GmbH, Mittelstetter Weg 2, D-86830 Schwabmünchen, Germany

## Abstract

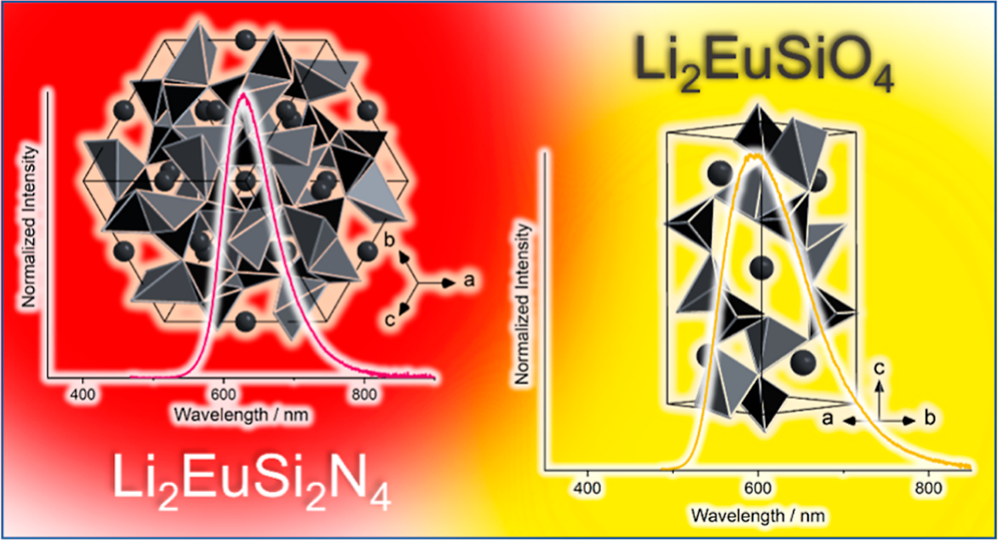

With Li_2_EuSi_2_N_4_, the end member of the solid-solution series set up by substituting the divalent cation in the well-known host materials Li_2_CaSi_2_N_4_ and Li_2_SrSi_2_N_4_ with Eu(II) could be synthesized and described by means of single-crystal X-ray diffraction and single-grain luminescence spectroscopy. The new compound crystallizes isotypically to Li_2_CaSi_2_N_4_ and Li_2_SrSi_2_N_4_ in cubic space group *Pa*3̅ (no. 205) with a lattice parameter of *a* = 10.7049(2) Å and a cell volume of *V* = 1226.73(7) Å^3^. Irradiated with blue light, Li_2_EuSi_2_N_4_ exhibits a narrow-band red emission (λ_max_ = 628 nm, 1.97 eV, 15924 cm^–1^; fwhm = 83 nm, 0.27 eV, 2143 cm^–1^). Also for Li_2_EuSiO_4_, the end member of the solid-solution series of Li_2_SrSiO_4_ with Sr(II) substituted by Eu(II), luminescence emission could be measured on a single crystal (λ_max_ = 595 nm, 2.05 eV, 16798 cm^–1^; fwhm = 116 nm, 0.40 eV, 3211 cm^–1^). In this paper, both compounds are being compared with other known members of their solid-solution series regarding structure and luminescence properties.

## Introduction

The most common approach to obtain solid-state luminescent materials, e.g., for use in light-emitting diodes (LEDs), is by combining a host structure with a doping agent. The requirements for suitable host structures are, besides the presence of suitable doping sites, stability against air, humidity, and decomposition at operating temperatures of LEDs.^1,2^ The doping agent is demanded to be excited efficiently in the ultraviolet (UV) to blue spectral region and to emit light of longer wavelengths, if possible narrow-banded and tunable by adjusting the local surroundings of the activator ion.^3,4^ A very suitable and widely used candidate for this is the divalent europium ion. Due to its parity-allowed 4f^6^(^7^F)5d^1^ ↔ 4f^7^(^8^S_7/2_) transitions, it provides a host-dependent emission that is generally easy to excite with UV to blue light.^5,6^

Even though there are now quite a few examples of Eu(II) being incorporated at cation sites with different valence and ionic radii or even at voids, the most obvious possibility is the substitution of divalent cations of a similar size, such as strontium.^7,8^ If this is the case, different substitution concentrations can be considered as steps of a solid-solution series starting with the pure host structure and leading toward a, in some cases only hypothetical, end member. The well-known compound Eu_2_Si_5_N_8_, for example, can be regarded as the end member in the substitution series of Eu in Sr_2_Si_5_N_8_ or Ba_2_Si_5_N_8_. The compound Eu[LiAl_3_N_4_] is in contrast to this only a hypothetical end member of a solid-solution series with Na[Li_3_SiO_4_] as the widely reported other end member.^9−11^

As the degree of substitution increases with the concentration of the activator ion, two phenomena are frequently observed. First, the existence of an emission-intensity maximum, usually for the lower single-digit percentage range as an optimum between available activator ions for energy conversion and the avoidance of concentration quenching at higher substitution levels.^12,13^ Second, a red shift of the emission peak, which is caused by self-absorption of converted photons on the high-energy side of the emission spectrum within the luminescent material based on additional activator ions on the light’s pathway.^14,15^

As host materials, lithosilicates have drawn some attention in the last five years. The term “lithosilicate” goes back to Rudolf Hoppe and refers to silicates which, in addition to silicon, also contain tetrahedrally coordinated lithium cations and thus allow a structural description based on the framework of these two different types of tetrahedra. The first representatives of alkali lithosilicates, such as K_3_LiSiO_4_, in which higher-coordinated metal ions are incorporated as completing cations in the aforementioned anionic tetrahedron structure, were synthesized and described by Hoppe’s working group as early as the 1980s.^16,17^ Due to their variety of shapes and rigid tetrahedron network as backbone with the possibility to incorporate various completing cations, this substance class has been rediscovered as a host structure for luminescence materials. Especially those representatives with relation to the UCr_4_C_4_-structure type exhibit promising emission properties.^16,18^

Here, a nitridic europium lithosilicate and an oxidic europium lithosilicate are presented. Interestingly, although the new phase Li_2_EuSi_2_N_4_ fulfills the formal composition rule for UCr_4_C_4_-related compounds, it is not structurally related to them. It is described based on single-crystal X-ray diffraction and luminescence spectroscopy. The same methodology is applied to the known phase Li_2_EuSiO_4_, which to date lacked reports on its luminescence.^19,20^

## Results and Discussion

**Figure 1:**
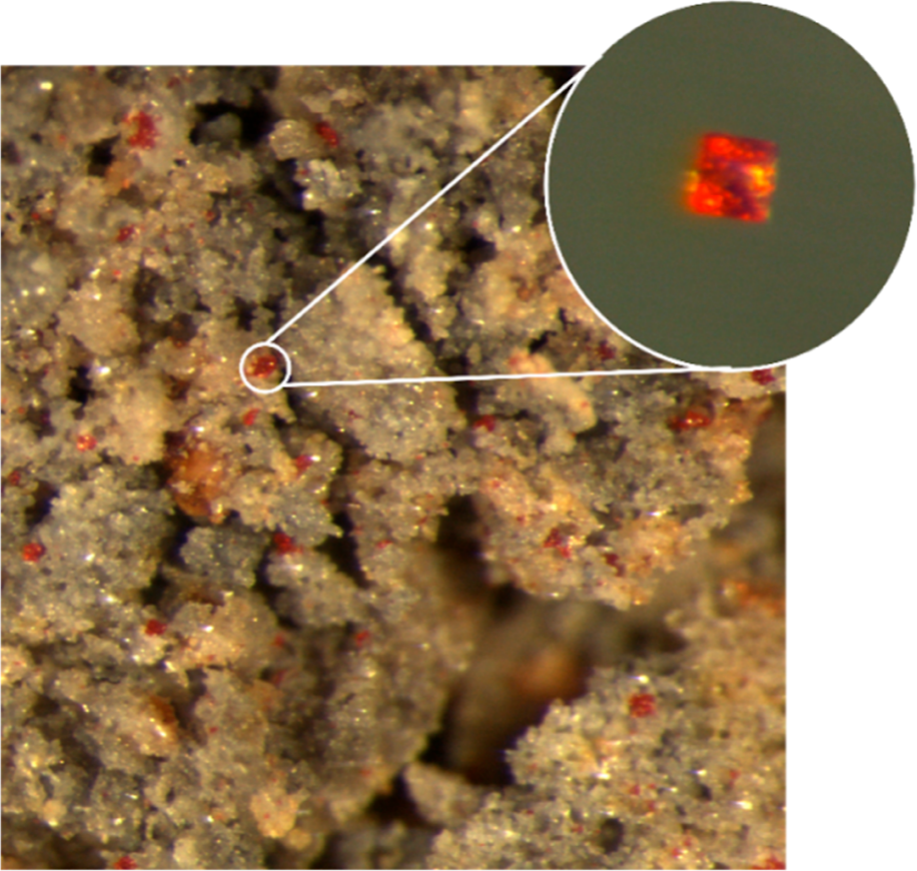
Light microscope image of a powder sample with Li_3_SiNO_2_ as the main phase and embedded crystals of Li_2_EuSi_2_N_4_. Inset: Single crystal of Li_2_EuSi_2_N_4_.

As can be seen in Figure 1, the new compound Li_2_EuSi_2_N_4_ forms crystals of octahedral shape with bright red body color, which are homogeneously distributed but easily distinguishable from the surrounding beige colored main phase Li_3_SiNO_2_.^8,21^ Several crystals have been isolated and measured via X-ray diffraction. The measurement and refinement details of the data set with the best *R*-values are listed in Table 1. Further information can be found in the Supporting Information: Wyckoff positions, atomic coordinates, and equivalent isotropic displacement parameters are listed in Table S1, anisotropic displacement parameters are listed in Table S2, and interatomic distances are listed in Table S3.

**Table 1:** Crystallographic Data and Structure Refinement of Li_2_EuSi_2_N_4_

parameter	value
empirical formula	Li_2_EuSi_2_N_4_
molar mass/g·mol^–1^	278.04
crystal system	cubic
space group	*Pa*3̅ (no. 205)
single-crystal diffractometer	Bruker D8 QUEST PHOTON III C14
radiation	Mo–K–L_2,3_ (λ = 0.71073 Å)
*a*/Å	10.7049(2)
*V*/Å^3^	1226.73(7)
formula units per cell Z	12
calculated density/g cm^–3^	4.52
crystal size/mm	0.040 × 0.040 × 0.040
temperature/K	278
absorption coefficient/mm^–1^	15.8
F(000)/e	1500
θ-range/deg	3.81–40.2
range in *hkl*	–19 < *h* < 19; −19 < *k* < 19; −19 < *l* < 19
reflections total/independent	73637/1204
*R*_int_	0.0364
reflections with I ≥ 2σ(I)	1089
*R*_σ_	0.0076
data/ref parameters	1204/42
absorption correction	multiscan (SADABS-2016/2)^22^
goodness of fit on F^2^	1.09
final *R*_1_/*wR*_2_ [*I* ≥ 2σ(*I*)]	0.0124/0.0290
final *R*_1_/*wR*_2_ (all data)	0.0151/0.0295
largest diff. peak/hole/e·Å^–3^	0.98/–1.23

**Figure 2:**
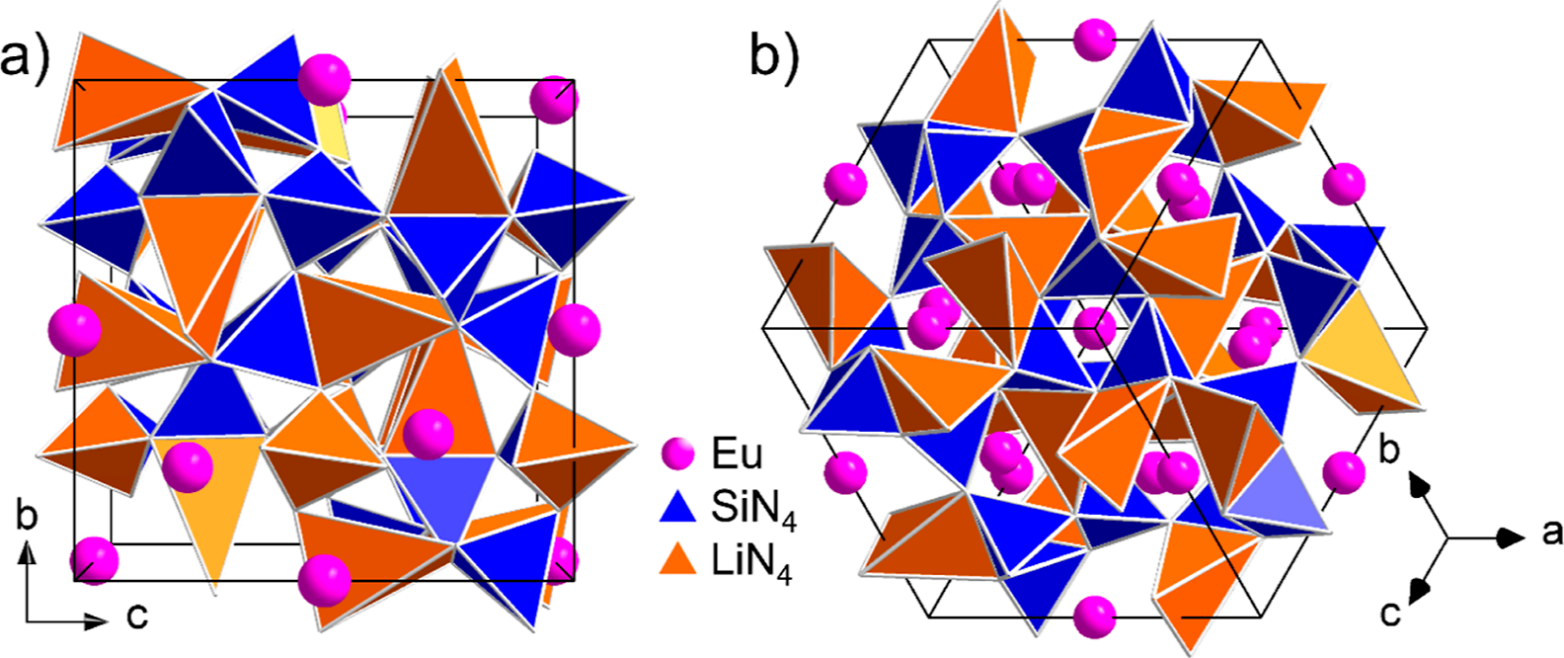
Crystal structure of Li_2_EuSi_2_N_4_ with the unit cell drawn in and seen (a) along the crystallographic *a*-axis and (b) in the [111] direction. All 4-fold coordinated cations are shown as tetrahedra. The nitrogen atoms at the tetrahedron corners are not shown for improved clarity.

Li_2_EuSi_2_N_4_ crystallizes in cubic space group *Pa*3̅ (no. 205) with a lattice parameter of *a* = 10.7049(2) Å and a cell volume of *V* = 1226.73(7) Å^3^. It is isotypic to the compounds Li_2_SrSi_2_N_4_ and Li_2_CaSi_2_N_4_ published by Zeuner et al., who also mentioned the existence of Li_2_EuSi_2_N_4_ in his dissertation, however, without publishing it in a journal or database. In particular, since Sr is always present in his syntheses, it cannot be ruled out that Sr(II) is also incorporated and influences the luminescence emission, which is suggested by the deviating measurement data.^19,23^ The unit cell, as can be seen in Figure 2, consists of 12 formula units. The structure has one silicon and one lithium position, as well as two crystallographically distinguishable positions each for europium and nitrogen.

The nitridolithosilicate can be described as a tectosilicate of corner-sharing [SiN_4_] tetrahedra. The lithium cation is also tetrahedrally coordinated by nitrogen anions and forms a corner-linked network as well. Taken together, there is a highly condensed, mainly corner-sharing tetrahedron network, where one [SiN_4_] and one [LiN_4_] tetrahedron are always linked also by a common edge. The degree of condensation is κ = 1 since, in contrast to Zeuner et al., a more general definition is used here, in which tetrahedrally coordinated lithium atoms are also considered as network formers and are included in the calculation of the degree of condensation.

**Figure 3:**
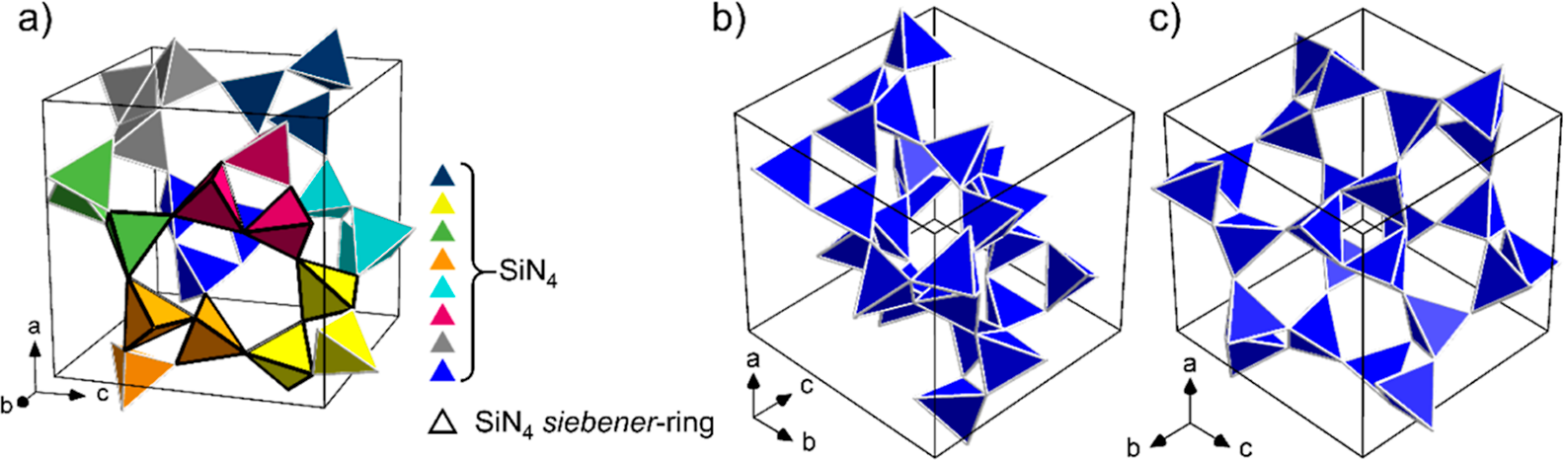
Silicate substructure of Li_2_EuSi_2_N_4_ with the unit cell drawn in, (a) with *dreier* rings in different colors and a selected *siebener* ring (black edges of the tetrahedra), (b) along the space diagonal [], and (c) along the space diagonal [111]. The nitrogen atoms at the tetrahedron corners are not shown for improved clarity.

The silicate substructure of the unit cell consists of a total of 24 corner-linked [SiN_4_] tetrahedra grouped into eight *dreier* rings,^24^ which in turn are linked by the corners of the resulting triangular shape. In this way, six *siebener* rings are formed, each of which is built from the tetrahedra of four *dreier* rings. Conversely, each [SiN_4_] tetrahedron is part of one *dreier* ring but also of several *siebener* rings. The silicate network and its constituting rings are shown from three different perspectives in Figure 3.

If only one unit cell is considered, then the silicate network appears as a layer parallel to the (111) plane. This is also visible in Figure 3b, where the line of sight is in the plane of the layer, and in Figure 3c, it is perpendicular to it. This results in two different types of space diagonals of the unit cell, three of which are in the plane of the layer, and one is outside, as can be seen in Figure S3.

**Figure 4:**
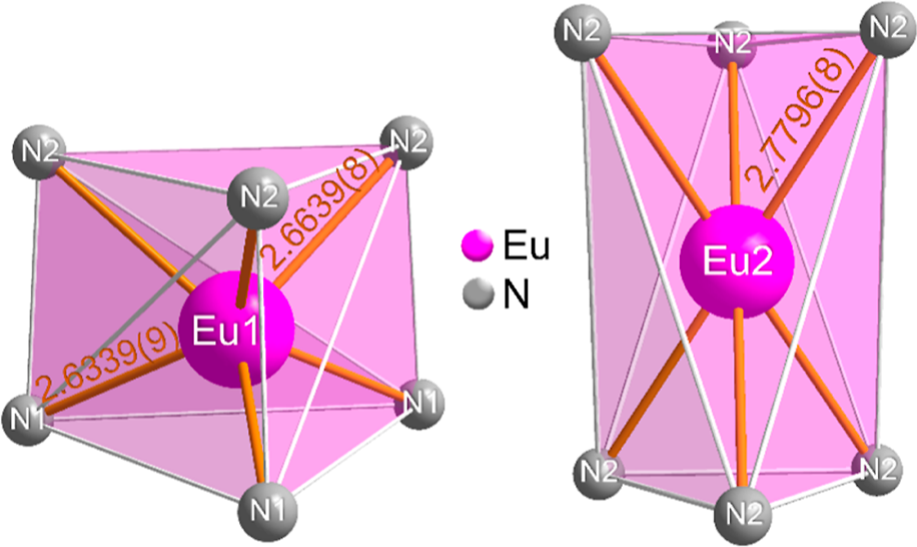
Coordination polyhedra of the two europium positions in Li_2_EuSi_2_N_4_ and associated bond lengths/Å to the nitrogen atoms.

The two europium positions are each 6-fold coordinated by nitrogen, with trigonal-prismatic coordination for Eu1 and trigonal-antiprismatic coordination for Eu2. In Figure 4, both coordination polyhedra are shown with the corresponding bond lengths.

Zeuner et al. and Wu et al. described the tetrahedron network as consisting only of [SiN_4_] tetrahedra with channels containing lithium and, in their case, calcium or strontium.^15,19^ However, since the coordination polyhedra of the ions in the channel are only linked by common corners and edges alternately, it seems more appropriate to denote them as a chain. This enables a differentiation to the channels with face-linked polyhedra as they occur, for example, in compounds with relation to the UCr_4_C_4_-structure type.^18^

Table 2 shows the most important differences between the new compound Li_2_EuSi_2_N_4_ and the already known compounds Li_2_SrSi_2_N_4_ and Li_2_CaSi_2_N_4_. As can be seen, the lattice parameter *a* correlates well with the ionic radii of the 6-fold coordinated species [100 pm for Ca(II), 117 pm for Eu(II), 118 pm for Sr(II)^25,26^]. Thus, from calcium via europium to strontium, the ionic radius, consequently the distance to the bonding partners, and ultimately the lattice parameter *a* increase.

**Table 2:** Comparison of Some Key Properties of Li_2_EuSi_2_N_4_, Li_2_CaSi_2_N_4_, and Li_2_SrSi_2_N_4_

	Li_2_CaSi_2_N_4_^19^	Li_2_EuSi_2_N_4_	Li_2_SrSi_2_N_4_^19^
molecular weight in g·mol^–1^	166.16	278.04	213.7
cell parameter *a* in Å	*a* = 10.569(12)	*a* = 10.7049(2)	*a* = 10.7137(12)
*V* in Å^3^	*V* = 1180.7(2)	*V* = 1226.73(4)	*V* = 1229.8(2)
	@ 295 K	@ 173 K	@ 293 K
body color	colorless	red	colorless
average distance M-N, in Å	2.528(3)	2.693(1)	2.699(4)
volume [MN_6_]-trigonal prism, in Å^3^	16.538	18.233	18.306
volume [MN_6_]-trigonal antiprism, in Å^3^	18.949	21.404	21.790
sum of ionic radii IR = *r*_M_ + *r*_N_, in Å^26^	2.46	2.63	2.64
IR,^3^ in Å^3^^26^	14.887	18.191	18.340

**Figure 5:**
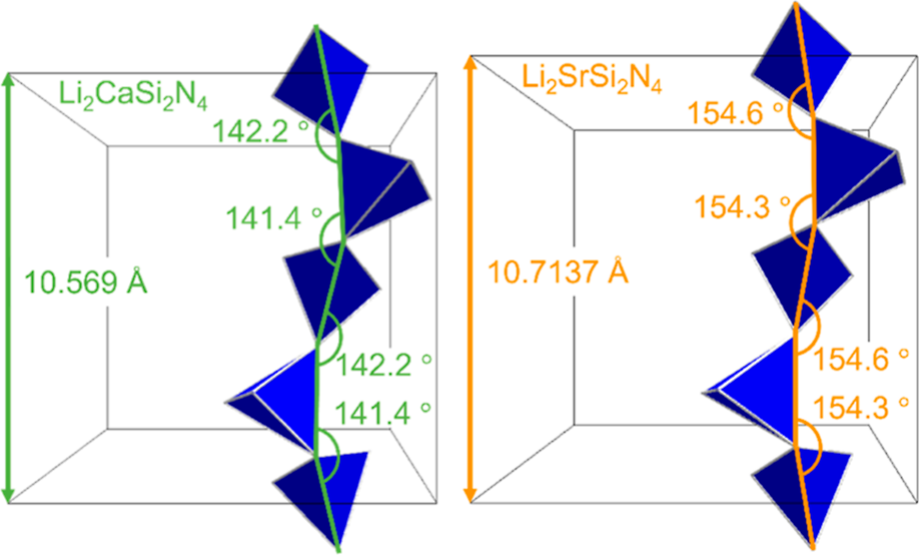
Comparison of the cell parameters and N–N–N-angles between chains of [SiN_4_] tetrahedra (blue) across the unit cells of Li_2_CaSi_2_N_4_ and Li_2_SrSi_2_N_4_.^19^

On the example of one strand of [SiN_4_] tetrahedra in Li_2_CaSi_2_N_4_ and Li_2_SrSi_2_N_4_, Figure 5 shows how the relatively rigid and highly condensed network can accommodate for the different-sized cations with a change in the N–N–N-angles. By this, a differing lattice parameter *a* is obtained despite the more or less unchanged Si–N distances and [SiN_4_] tetrahedron volumes, respectively.

Besides the structural comparison, one regarding the luminescence properties of the new compound with other members of the solid-solution series is also of interest. Therefore, luminescence spectroscopy measurements were carried out on single crystals of Li_2_EuSi_2_N_4_.

**Figure 6:**
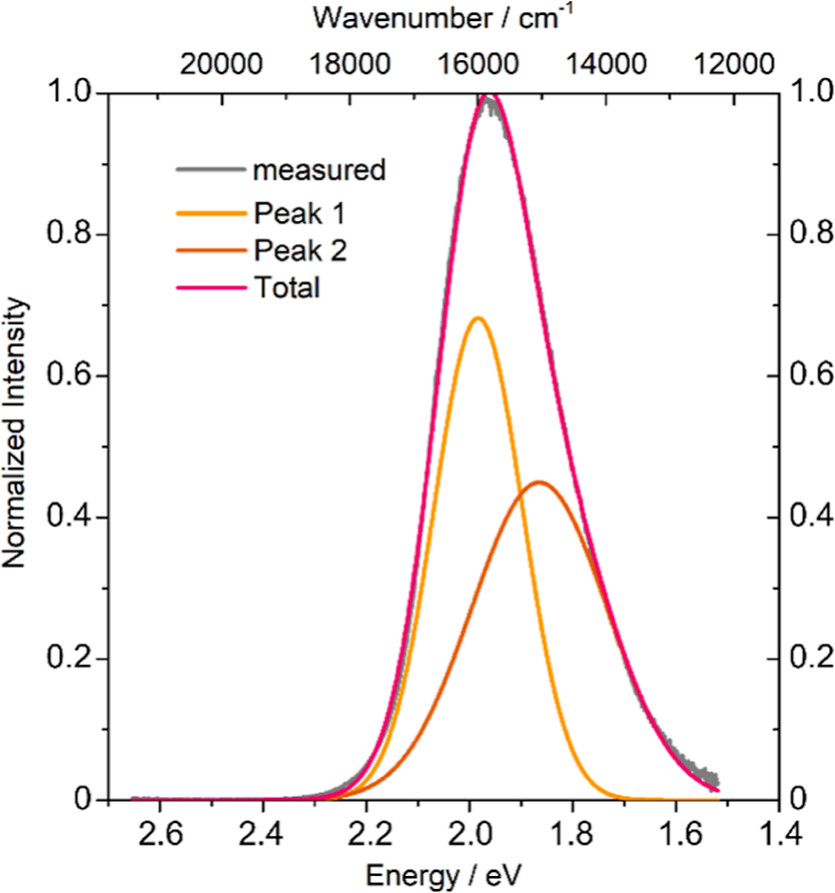
Emission spectrum of a single crystal of Li_2_EuSi_2_N_4_ upon excitation with blue light (λ_exc_ = 448 nm) with bimodal Gauss decomposition on the wavenumber and energy scale.

As can be seen in Figure 6, the luminescence maximum upon excitation with a 448 nm laser is located in the red spectral region at λ_max_ = 628 nm (1.97 eV, 15924 cm^–1^) with a full-width at half-maximum (fwhm) of the emission band of 83 nm (0.27 eV, 2143 cm^–1^). Corresponding to the two positions of Eu(II) in the compound, the emission spectrum on the energy and wavenumber scale of Li_2_EuSi_2_N_4_ can be decomposed into two Gauss curves. Further details can be found in Table S4 in the Supporting Information.

For the isotypic compound Li_2_CaSi_2_N_4_:Eu^2+^, Wu et al. found the emission peak to be increasing from 590 to 594 nm with rising Eu(II) content (0.5 to 2 mol % relative to Ca content) with also increasing fwhm from 85 to 94 nm (λ_exc_ = 400 nm).^27^ Similarly for Li_2_SrSi_2_N_4_:Eu^2+^, an emission peak shift from 613 to 620 nm with increasing Eu(II) content (0.5 to 3 mol % relative to Sr content) was reported by Wu et al. along with a fwhm of 100 nm at a 615 nm peak wavelength (λ_exc_ = 395 nm).^15^ As only single crystals and no powder sample of Li_2_EuSi_2_N_4_ are available to date, no excitation spectrum could be generated. Nevertheless, it can be assumed that the spectrum is very similar to those of the isotypic phases. Here, a single band with excitation maxima of 400 nm for Li_2_CaSi_2_N_4_:Eu^2+^ and 395 nm for Li_2_SrSi_2_N_4_:Eu^2+^ has been reported.^15,27^

The differences in emission wavelengths of the Ca and Sr compounds appear counterintuitive at first glance. Normally we would expect that the activator ion in the smaller Ca position experiences stronger interaction with its ligands, leading to increased crystal-field splitting and thereby to a red-shifted emission. In contrast to that is the reported blue-shifted emission of Li_2_CaSi_2_N_4_:Eu^2+^ compared with that of Li_2_SrSi_2_N_4_:Eu^2+^. Interestingly, to our knowledge, there is so far no explanation in the literature for possible reasons for the deviation of the emission peak positions from the above-mentioned intuitive chain of reasoning.^15,27^

One possible approach to explain the different emission properties of the three isotypic phases is presented in the following. Despite the fact that we have no direct information about the local environment of the Eu(II) ions and the position in the structure can only be assumed, we can try to derive trends from the comparison of the average structures from single-crystal X-ray diffraction data. Assuming further that the host structure is mainly stabilized by the anionic substructure including defined metal–ligand polyhedra, the metal–ligand bond lengths around an activator ion should remain almost unchanged upon incorporation of the activator in the low percentage range, and thus, no dramatic local structure relaxation occurs. In the following, the observed structural trends are put in relation to the luminescence properties.

When the sum of the ionic radii is compared with the actual metal–nitrogen bond length from the average structure model,^28^ for Ca–N, the effective ionic radii sum up to 2.46 Å for 6-fold coordinated Ca^2+^ and 4-fold coordinated N^3–^, whereas for Sr–N and a likewise coordination, a sum of 2.64 Å is yielded.^26^ Since the average *M*−N bond lengths in the Li_2_*M*Si_2_N_4_ compounds are 2.528(3) Å for *M* = Ca and 2.699(4) Å for *M* = Sr, it becomes clear that the actual metal–nitrogen distances are not maximally shortened, which causes a prestrained lattice.^29^ This being said, it is of particular interest that for the Ca compound, the deviation relative to the ionic radius sum is even greater. Assuming the amount of this prestrain or more generally the difference between the theoretical spatial requirements given by the ionic radii sum and the bond lengths in the compound as a relevant factor for the behavior of an incorporated activator ion, this relatively bigger difference would lead to a weaker Eu-ligand interaction in Li_2_CaSi_2_N_4_:Eu^2+^, thus to a smaller crystal-field splitting and, with this, to a blue-shifted emission compared to the Sr compound, where the difference between the ionic radii sum and present metal–ligand distance is smaller.^30^

**Figure 7:**
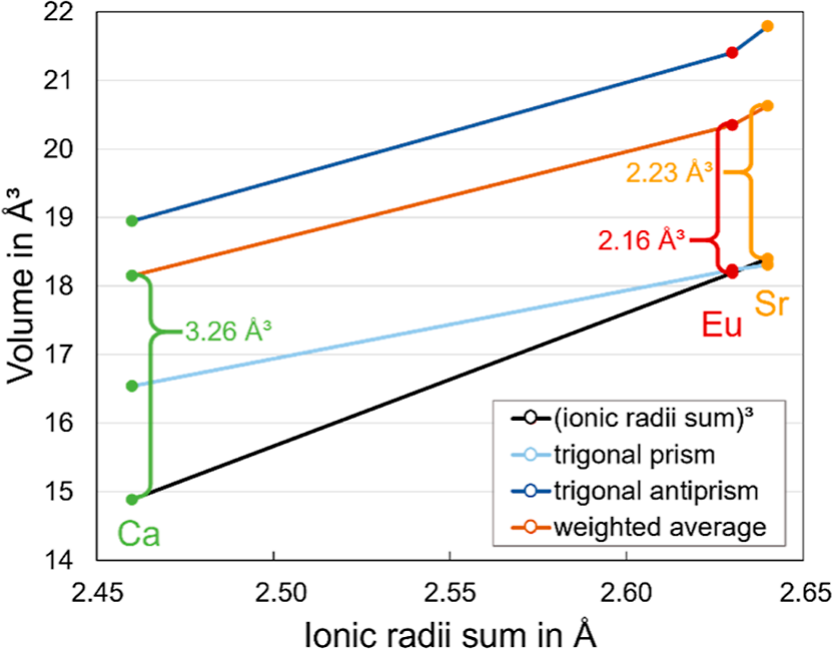
Plot of the [*M*N_6_] polyhedral volumes in Li_2_*M*Si_2_N_4_ (*M* = Ca, Eu, and Sr) and the cubes of the ionic radii sum (IR_*M*_ + IR_N_)^3^ against the ionic radii sum IR_*M*_ + IR_N_. “Weighted average” means the average polyhedron volume considering the frequency of occurrence of the different polyhedra.

This discrepancy becomes even more pronounced if coordination polyhedra are considered. If one compares (as displayed in Figure 7) the polyhedral volumes of the 6-fold-coordinated species in the isotypic compounds Li_2_*M*Si_2_N_4_ (*M* = Ca, Eu, and Sr) with the cube of the ionic radii sum as a reference, it becomes obvious that the polyhedra around Ca (despite being the smallest in absolute numbers) are the largest regarding the steric requirements of the centering cation. This implies for the Ca compound less interaction of the activator with its ligands compared to the Sr compound in relation to the absolute cation’s size and thus leads to a less red-shifted emission or, in other words, that Li_2_CaSi_2_N_4_:Eu^2+^ emits (surprisingly at first glance) at higher energies than Li_2_SrSi_2_N_4_:Eu^2+^. Although the differences are very small comparing the Sr with the Eu compound, the explanatory approach given above may also be valid here, as Figure 7 indicates.

For Li_2_EuSi_2_N_4_, the red-shifted yet narrower emission can additionally be explained by an increased self-absorption due to the significantly increased number of activator ions. This assumption for the mechanism of the red shift is supported by the fact that the fwhm of the emission decreases significantly compared with the Ca and Sr isotypes as it can be seen even on the wavelength scale.^31^

The reported initial broadening of the emission peak with increasing Eu(II) content in Li_2_CaSi_2_N_4_:Eu^2+^ may be due to the population of the nonpreferred Ca site, which is presumably the smaller prismatic coordinated one.^23^ In contrast to this stands the aforementioned narrower emission band of the fully occupied end member Li_2_EuSi_2_N_4_. This indicates that, at higher substitution levels, the effects of self-absorption outride the increase of the half-width caused by the superposition of the two emission bands originating from the two crystallographically different activator sites.

Like Li_2_EuSi_2_N_4_ can be seen as the fully europium-occupied end member of the nitridosilicate phosphors Li_2_CaSi_2_N_4_:Eu^2+^ and Li_2_SrSi_2_N_4_:Eu^2+^, it is Li_2_EuSiO_4_ for the oxosilicate phosphor Li_2_SrSiO_4_:Eu^2+^. Unlike the first title compound, Li_2_EuSiO_4_ has been described before by Haferkorn et al. in 1998 based on single-crystal X-ray diffraction data. However, no luminescence measurements have been reported. Meanwhile, the isotypic compound Li_2_SrSiO_4_, also first described by Haferkorn et al., has been successfully doped with Eu(II) by several working groups yielding emissions in the yellow spectral region.^20,32−34^

Since orange single crystals of Li_2_EuSiO_4_ were found as side phases in different samples, it was possible to determine the crystal structure and emission properties of one single-crystalline individual, thus closing this gap in the literature. The measurement and refinement details of the single-crystal X-ray diffraction are listed in the Supporting Information in Table S5. Wyckoff positions, atomic coordinates, and equivalent isotropic displacement parameters are listed in Table S6, anisotropic displacement parameters in Table S7, and interatomic distances in Table S8.

**Figure 8:**
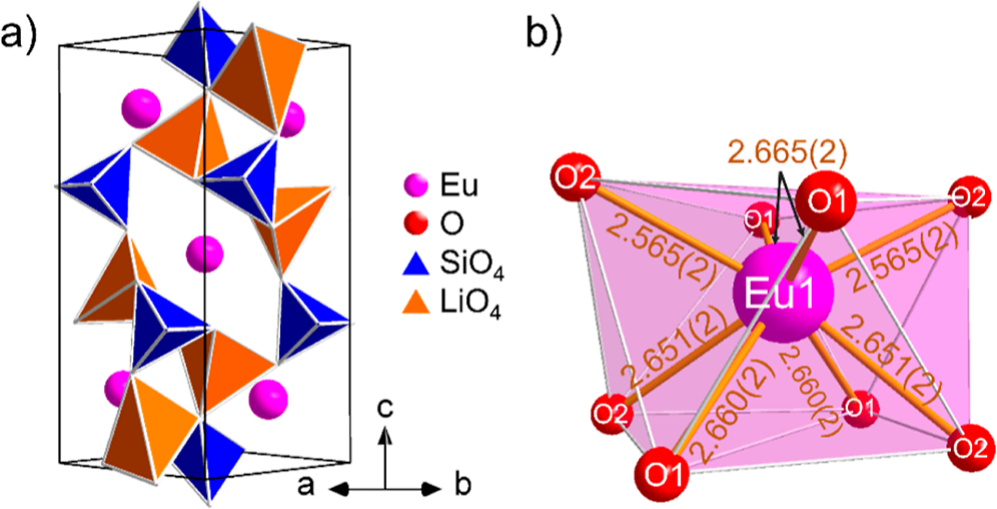
(a) Crystal structure of Li_2_EuSiO_4_ with the unit cell drawn in and seen in the [] direction. The oxygen atoms at the tetrahedron corners are not shown for improved clarity. (b) Coordination polyhedron of the europium atom with the corresponding bond lengths to the oxygen atoms in Å.

As shown in Figure 8a, the structure of Li_2_EuSiO_4_ consists of a corner-sharing lithosilicate network with isolated [SiO_4_] tetrahedra, allowing the classification also as an orthosilicate.^35^ The anionic tetrahedron network is filled with the 8-fold coordinated europium ions. The coordination polyhedron, which is visible in Figure 8b, can be described as a distorted square antiprism or double-capped trigonal prism. The degree of condensation is κ = 0.75 since both silicon and lithium are tetrahedrally coordinated and counted as network formers.

**Figure 9:**
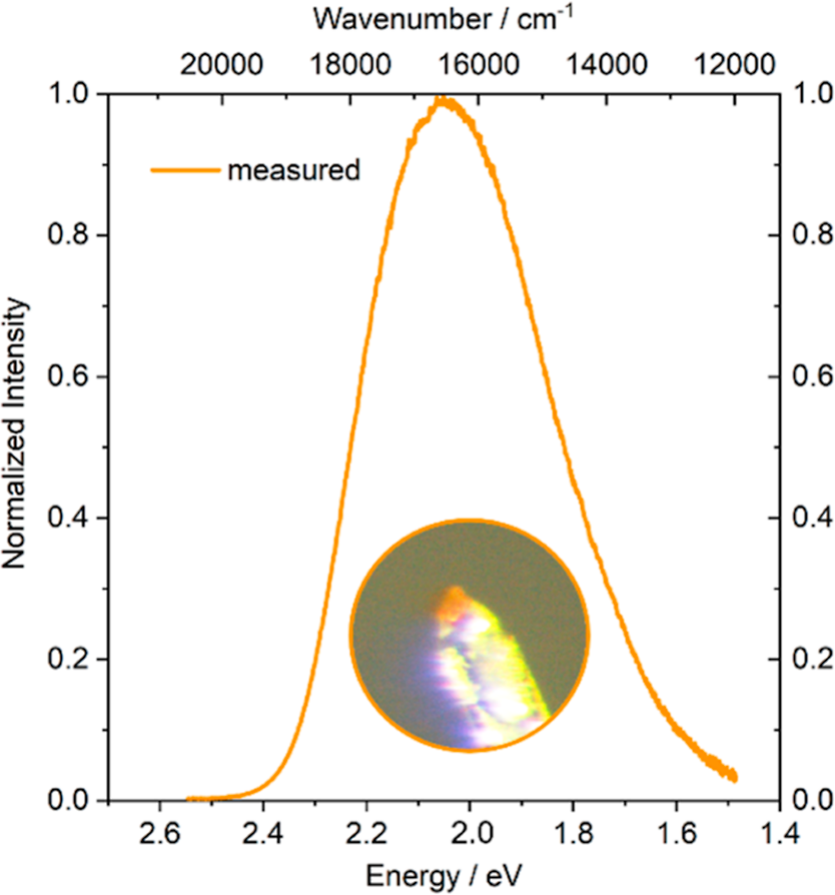
Emission spectrum of a single crystal of Li_2_EuSiO_4_ upon excitation with blue light (λ_exc_ = 448 nm). Inset: An orange single crystal of Li_2_EuSiO_4_ prepared on a glass pin for X-ray diffraction.

Obviously, a full occupation of the divalent cation position with europium represents not the ideal state for luminescence performance since concentration quenching is to be expected. Nevertheless, as the isotypic compound Li_2_SrSiO_4_ is known for its luminescence when doped with Eu(II), also for the fully occupied end member, an emission is very likely. Luminescence spectroscopy of the above-described single crystal yielded an emission peak of λ_max_ = 595 nm (2.05 eV, 16798 cm^–1^) with a fwhm of 116 nm (0.40 eV, 3211 cm^–1^) under irradiation with blue light (λ_exc_ = 448 nm). In Figure 9, the measured single crystal and the obtained emission spectrum can be seen. As no powder sample of Li_2_EuSiO_4_ was synthesized, an excitation spectrum could not be generated. For Li_2_SrSiO_4_:Eu^2+^, a double band excitation spectrum with maxima around 300 nm and around 400 nm has been reported, so similar values can be expected for Li_2_EuSiO_4_.^32^

Compared to the Eu(II)-doped Sr isotype, for which emission maxima of λ_max_ = 560–580 nm with fwhm around 100 nm have been reported, we see a red shift of the luminescence for the fully occupied end member.^32,33^ As the volumes of both the unit cell (*V*_Sr_ = 272.8 Å^3^, *V*_Eu_ = 272.91 Å^3^)^20^ and the polyhedron around the divalent cation (*V*_Sr[EuN8]_ = 30.52 Å^3^, *V*_Eu[EuN8]_ = 30.56 Å^3^)^20,36^ are very similar for the two compounds, the interaction strength of Eu(II) with its ligands can hardly be used as an explanation for the observed shift. As described above for Li_2_EuSi_2_N_4_, the red shift can be explained by the occurrence of self-absorption of converted high-energy photons by other Eu(II) ions, which affects the high-energy side of the spectrum and by this causes the peak to shift to longer wavelengths.^31^ Since the tetrahedron network of Li_2_EuSiO_4_ is not as condensed and rigid as in Li_2_EuSi_2_N_4_, one can also think of an effect of the electronic excitation on the structure, which could cause line broadening in the emission due to different deformations in the local environment of the activated Eu(II) ions.

Since we have now described two luminescent europium lithosilicates, the question arises as to what causes their different luminescence properties. The Eu(II) ion is located in significantly different environments in the two structures. In Li_2_EuSi_2_N_4_, we see two highly symmetric 6-fold coordinated sites with nitrogen as the ligand, whereas in Li_2_EuSiO_4_, the ligand is oxygen, and the single site is rather distorted 8-fold coordinated. From the lower coordination number, the smaller polyhedron volume, and the higher valent ligand, we can derive a much more pronounced nephelauxetic effect and a greater interaction of the Eu(II) ion in the nitridic compound, which causes a larger crystal-field splitting and, by this, an emission shifted to longer wavelengths. However, the emission of Li_2_EuSi_2_N_4_ is not only red-shifted with respect to Li_2_EuSiO_4_, but it is also significantly narrower, even though a superposition of the emissions of two different activator sites is present. This narrow emission can probably be attributed to the more condensed tetrahedra network (κ_Li2EuSi2N4_ = 1 > κ_Li2EuSiO4_ = 0.75) and the greater structural rigidity of the tectosilicate compared to the orthosilicate, as well as the higher symmetry in the environment.^7,37^

## Conclusions

With this contribution, the emission properties (λ_exc_ = 448 nm) of Li_2_EuSiO_4_ and Li_2_EuSi_2_N_4_ as well as the single-crystal structure of the latter compound have been reported for the first time.

The europium nitridolithosilicate Li_2_EuSi_2_N_4_ was identified as a minor phase of several syntheses. The existence of single crystals of sufficient size and excellent quality allowed the structure to be solved, refined, and to place the found phase in a row with its known isotypes Li_2_SrSi_2_N_4_ and Li_2_CaSi_2_N_4_. Since these compounds, synthesized and published for the first time by Zeuner et al.,^19^ show luminescence when doped with Eu(II), also for Li_2_EuSi_2_N_4_, luminescence spectroscopy has been conducted, yielding a narrow band red luminescence λ_max_ = 628 nm (1.97 eV, 15924 cm^–1^) with a fwhm of 83 nm (0.27 eV, 2143 cm^–1^) under irradiation with blue light (λ_exc_ = 448 nm).

Attempts have been made to explain the differences in the emission properties of the three isotypic compounds, with differences in the local environment of the activator sites. For this purpose, a comparison of polyhedron volumes and fictive reference volumes based on the sum of the effective ionic radii has been drawn in order to assess the interaction strength between cations and their ligand field. This concept may also prove useful in other cases, where the cation size and luminescence shift behave counterintuitively at first glance. In isotypic structures, one would expect a blue-shifted Eu(II)-based emission for the phase hosting the bigger cation due to a larger average polyhedron volume and less interaction of Eu(II) with the ligands. This is the case, e.g., for CaAlSiN_3_:Eu^2+^ (CASN) and SrAlSiN_3_:Eu^2+^ (SASN) or the phosphors of the 258-type (M_2-x_Eu_x_Si_5_N_8_, M = Sr and Ba). In contrast to that are Y_3_Al_5_O_12_:Ce^3+^ (YAG) and Lu_3_Al_5_O_12_:Ce^3+^ (LuAG) as another pair of isotypic structures, where the observed luminescence is not in accordance to the expectations based solely on the ionic radius but according to the above-described model for the title phase Li_2_EuSi_2_N_4_.^9,10,38−46^

Also found as a minor phase, the europium lithosilicate Li_2_EuSiO_4_ allowed the determination of structure and emission properties on the same individual due to its single-crystalline form. While the structure was reported before by Haferkorn et al.,^20^ the luminescence [λ_max_ = 595 nm (2.05 eV, 16798 cm^–1^), fwhm = 116 nm (0.40 eV, 3211 cm^–1^), and λ_exc_ = 448 nm] is presented for the first time.

Compounds like the ones presented in this work are not particularly of interest for commercial applications due to issues like high costs and severe concentration quenching, both caused by the high europium content. Nevertheless, they are very valuable from a scientific point of view to gain a better understanding of the structure and luminescence behavior of rare-earth-substituted phosphors through the role of Li_2_EuSi_2_N_4_ and Li_2_EuSiO_4_ as extreme examples of a full substitution.

## Experimental Section

### Synthesis of Li_2_EuSi_2_N_4_

Using a high-temperature solid-state reaction in arc-welded tantalum tubes under argon atmosphere, it was possible to obtain single crystals as side phases from the starting materials Si_3_N_4_ (UBE SN-E10, >99.9%) and Li_2_O (Sigma-Aldrich, 97%) with a stoichiometric ratio of 1:4. The powders were weighed and mixed along with 5 wt % LiF (Sigma-Aldrich, >99.99%) as flux and 6 mol % EuF_2_ (Alfa Aesar, 99.9%) using an agate mortar. The tantalum tubes were placed inside silica glass ampules containing a low-pressure inert gas atmosphere (Ar 5.0, Messer Austria GmbH). The temperature profile of the synthesis consists of a heating ramp of 240 K/h up to 900 °C, a dwelling time of 20 h, and subsequently a cooling ramp of −6 K/h to 150 °C. Afterward, the furnace is switched off, and once cooled to room temperature, the samples can be handled under air without detectable decomposition in several months.

### Synthesis of Li_2_EuSiO_4_

Since the single crystal of Li_2_EuSiO_4_ described above was obtained only accidentally as the side phase, no synthesis protocol will be given here. Instead, reference is made to the description which has been published by Haferkorn et al.^20^ It should be noted that no source of strontium was used in the synthesis.

### Microscopy

A Leica M125 stereomicroscope with the external light sources SCHOTT KL1500 LCD and KL2500 LCD and a Leica DFC420 camera has been used for sample examination and the preparation of single crystals.

### Single-Crystal X-ray Diffraction

For structural investigations based on single crystals, a Bruker D8 QUEST diffractometer (Mo–K–L_2,3_ radiation, *l* = 0.71073 Å), equipped with a PHOTON III detector, was used. Multiscan absorption correction and data processing were done using the SAINT^47^ and SADABS^22^ software tools. The structure was solved using charge flipping implemented in Superflip;^48,49^ the refinement was done with Jana2006.^50^ Pictures of the crystal structure were generated using DIAMOND 4.6.4.^51^ Further crystallographic data can be found under the deposition numbers 2320923 (Li_2_EuSi_2_N_4_) and 2321183 (Li_2_EuSiO_4_) at the joint Cambridge Crystallographic Data Centre and Fachinformationszentrum Karlsruhe Access Structures Service www.ccdc.cam.ac.uk/structures.

### Luminescence Spectroscopy

To measure emission spectra of single crystals, a setup of a blue laser diode (λ = 448 nm, THORLABS, Newton, NJ, US) and a CCD detector (AVA AvaSpec 2048, AVANTES, Apeldoorn, Netherlands) in connection with the software AVA AvaSoft (version 7) was used (wavelength range recorded: 470.7–900.0 nm; step size: 0.33 nm).

## Supporting Information Available

Atomic coordinates, displacement parameters, and interatomic distances of Li_2_EuSi_2_N_4_; Gauss-decomposition parameters of the emission spectrum of Li_2_EuSi_2_N_4_; and crystallographic data, structure refinement, atomic coordinates, displacement parameters, and interatomic distances of Li_2_EuSiO_4_ (PDF)
