# Supplementary material for: Europium Lithosilicates Li2EuSi2N4 and Li2EuSiO4—Crystal Structures and Luminescence
Source: Chem Mater. 2024 Sep 12;36(19):9882–9. doi: 10.1021/acs.chemmater.4c02070 (PMC11468888; doi:10.1021/acs.chemmater.4c02070)
Supplement: Supplementary file 1 — cm4c02070_si_001.pdf [file cm4c02070_si_001.pdf]

Supporting Information

to

Europium Lithosilicates $Li_2EuSi_2N_4$ and Li_2EuSiO_4 – Crystal Structures and Luminescence

Kilian M. Rießbeck[a], Markus Seibald[b], Christiane Stoll[b], and Hubert Huppertz*[a]

[a] K. M. Rießbeck, Univ.-Prof. Dr. H. Huppertz:

Department of General, Inorganic and Theoretical Chemistry,

University of Innsbruck

Innrain 80-82, A-6020 Innsbruck, Austria

E-Mail: *Hubert.Huppertz@uibk.ac.at*

https://www.uibk.ac.at/en/aatc/ag-huppertz

[b] Dr. M. Seibald, Dr. C. Stoll:

ams-OSRAM International GmbH,

Mittelstetter Weg 2, D-86830 Schwabmünchen, Germany

Table S1: Wyckoff Positions, Atomic Coordinates, and Equivalent Displacement Parameters for $Li_2EuSi_2N_4$ (Standard Deviations in Parentheses)

Atom	Wyckoff-Position	x	*y*	*z*	U_{eq}
Eu1	8*c*	0.283947(5)	0.783947(5)	0.716053(5)	0.00560(2)
Eu2	4*b*	0	0	1/2	0.01273(2)
Si1	24*d*	0.25583(3)	0.97913(2)	0.36914(3)	0.00275(5)
N1	24*d*	0.37482(8)	0.93861(9)	0.26846(8)	0.0070(2)
N2	24*d*	0.22823(8)	0.87643(7)	0.49185(8)	0.0045(2)
Li1	24*d*	0.3928(3)	0.7723(3)	0.4699(3)	0.0148(6)

Table S2: Anisotropic Displacement Parameters U_{ij} ($Å^2$) of $Li_2EuSi_2N_4$ (Standard Deviations in Parentheses)

**Atom**	U_{11}	U_{22}	U_{33}	U_{23}	U_{13}	U_{12}
Eu1	0.00560(3)	0.00560(3)	0.00560(3)	-0.00041(2)	-0.00041(2)	0.00041(2)
Eu2	0.01273(3)	0.01273(3)	0.01273(3)	0.00526(2)	-0.00526(2)	0.00526(2)
Si1	0.00232(9)	0.00250(9)	0.00344(9)	-0.00005(7)	-0.00027(7)	-0.00028(7)
N1	0.0037(3)	0.0082(3)	0.0092(3)	0.0023(2)	-0.0013(2)	0.0013(2)
N2	0.0050(3)	0.0034(2)	0.0052(3)	-0.0008(2)	0.0003(3)	-0.0008(3)
Li1	0.0098(9)	0.024(2)	0.0107(9)	0.0023(7)	0.0013(8)	0.0044(8)

Table S3: Selected Interatomic Distances for $Li_2EuSi_2N_4$ in Å (Standard Deviations in Parentheses)

**Eu1**	**- N1**	2.6339(9)	×3		
	**- N2**	2.6639(8)	×3	Ø Eu2-N	2.649
**Eu2**	**- N2**	2.7796(8)	×6		
**Si1**	**- N1**	1.7240(9)			
	**- N1**	1.7366(9)			
	**- N2**	1.7381(9)			
	**- N2**	1.7634(8)		Ø Si1-N	1.741
**Li1**	**- N1**	2.361(3)			
	**- N1**	2.072(3)			
	**- N2**	2.098(3)			
	**- N2**	2.110(3)		Ø Li1-N	2.160

Table S4: Parameters of the Two Gauss-Curves Obtained by the Decomposition of the Single-Crystal Emission Spectrum on the Energy and Wavenumber Scale of $Li_2EuSi_2N_4$

	$Li_2EuSi_2N_4$ (λ_{exc} = 448 nm)	
	Peak 1	*Peak 2*
Rel. intensity	0.50	0.50
E_{peak} / eV	1.98	1.86
$\tilde{\nu}_{peak}$ / cm^{-1}	15994	15039
FWHM / eV	0.20	0.31
FWHM / cm^{-1}	1627	2484

Table S5: Crystallographic Data and Structure Refinement of Li_2EuSiO_4.

parameter	value
empirical formula	Li_2EuSiO_4
molar mass / g·mol^{-1}	257.9
crystal system	trigonal
space group	$P3_221$ (no. 154)
single-crystal diffractometer	Bruker D8 QUEST PHOTON III C14
radiation	Mo-K-$L_{2,3}$ (λ = 0.71073 Å)
a / Å	5.0316(3)
c / Å	12.4790(9)
V /Å^3	273.60(3)
formula units per cell Z	3
calculated density / g cm^{-3}	4.70
crystal size / mm	0.030 × 0.030 × 0.030
temperature / K	301
absorption coefficient / mm^{-1}	17.4
F(000) / e	345
θ-range / deg	4.68 - 42.07
range in hkl	$-9<h<9$; $-9<k<9$; $-23<l<23$
reflections total / independent	15972 / 1282
R_{int}	0.0295
reflections with $I \geq 2\sigma(I)$	1258
R_σ	0.146
data / ref. parameters	1282 / 38
absorption correction	multi-scan (SADABS-2016/2)[1]
goodness of fit on F^2	0.93
final R_1 / wR_2 [$I \geq 2\sigma(I)$]	0.0106 / 0.0267
final R_1 / wR_2 (all data)	0.0110 / 0.0269
largest diff. peak/hole / e·Å^{-3}	0.83 / -0.97

Table S6: Wyckoff Positions, Atomic Coordinates, Equivalent Displacement Parameters, and Occupations for Li_2EuSiO_4

**Atom**	**Wyckoff-Position**	**x**	***y***	***z***	U_{eq}
Eu1	3*b*	0.41732(2)	0.41732(2)	1/2	0.00732(2)
Si1	3*a*	0.2773(2)	0.2773(2)	0	0.0049(2)
Li1	6*c*	0.5926(7)	0.9268(7)	0.9208(2)	0.0115(9)
O1	6*c*	0.4835(2)	0.4499(3)	0.10609(7)	0.0080(3)
O2	6*c*	0.9328(2)	0.2388(2)	0.00341(8)	0.0078(3)

Table S7: Anisotropic Displacement Parameters U_{ij} (Å^2) of Li_2EuSiO_4 (Standard Deviations in Parentheses)

Atom	U_{11}	U_{22}	U_{33}	U_{23}	U_{13}	U_{12}
Eu1	0.00792(3)	0.00792(3)	0.00659(3)	-0.00044(1)	0.00044(1)	0.00431(3)
Si1	0.0051(2)	0.0051(2)	0.0047(2)	0.00003(6)	-0.00003(6)	0.0026(2)
Li1	0.011(2)	0.012(2)	0.010(2)	-0.0029(8)	-0.0019(8)	0.0047(9)
O1	0.0083(3)	0.0097(3)	0.0066(3)	-0.0021(3)	-0.0017(2)	0.0049(3)
O2	0.0056(3)	0.0088(3)	0.0085(3)	0.0001(2)	0.0002(2)	0.0033(3)

Table S8: Selected Interatomic Distances for Li_2EuSiO_4 in Å (Standard Deviations in Parentheses)

**Eu1**	**-O1**	2.660(2)	×2		
	**-O1**	2.665(2)	×2		
	**-O2**	2.565(2)	×2		
	**-O2**	2.651(2)	×2	Ø Eu1-O	2.635
**Si1**	**-O1**	1.6377(9)	×2		
	**-O2**	1.646(2)	×2	Ø Si1-O	1.642
**Li1**	**-O1**	1.965(3)			
	**-O1**	2.000(4)			
	**-O2**	1.942(3)			
	**-O2**	2.029(4)		Ø Li1-O	1.984

References

[1] L. Krause, R. Herbst-Irmer, G. M. Sheldrick, D. Stalke, *J. Appl. Crystallogr.* **2015**, *48*, 3–10.
